# High Prevalence of Pulmonary Tuberculosis but Low Sensitivity of Symptom Screening among HIV-Infected Pregnant Women in South Africa

**DOI:** 10.1371/journal.pone.0062211

**Published:** 2013-04-17

**Authors:** Christopher J. Hoffmann, Ebrahim Variava, Modiehi Rakgokong, Katlego Masonoke, Martin van der Watt, Richard E. Chaisson, Neil A. Martinson

**Affiliations:** 1 Center for TB Research, Johns Hopkins University School of Medicine, Baltimore, Maryland, United States of America; 2 Department of Medicine, Klerksdorp Tshepong Hospital Complex and University of the Witwatersrand, Klerksdorp, South Africa; 3 Perinatal HIV Research Unit, University of the Witwatersrand, Johannesburg, South Africa; San Francisco General Hospital, University of California San Francisco, United States of America

## Abstract

Symptom screening is a recommended component of intensified case-finding (ICF) for pulmonary tuberculosis (TB) among HIV-infected individuals. Symptomatic individuals are further investigated to either exclude or diagnose pulmonary TB, thus reducing the number of individuals requiring costly laboratory investigation. Those with laboratory evaluations negative for pulmonary TB or who lack symptoms may be eligible for antiretroviral therapy (ART) and/or TB isoniazid preventive therapy (IPT). A four-part symptom screen has been recommended by the World Health Organization (WHO) for identifying TB suspects and those unlikely to have TB. A meta-analysis of studies among HIV-infected individuals calculated a sensitivity of 90.1% for the four-part symptoms screen - of any of cough, fever, night sweats, or weight loss - among patients in clinical care, making it an effective tool for identifying most patients with TB. An important population for intensified case-finding not included in that meta-analysis was HIV-infected pregnant women. We undertook a cross-sectional survey among HIV-infected pregnant women receiving prenatal care at community clinics in South Africa. We obtained a four-symptom review and sputum smear microscopy and mycobacterial culture on all participants. Among 1415 women, 226 (16%) had a positive symptom screen, and 35 (2.5%) were newly diagnosed with culture-positive TB. Twelve were on TB treatment at the time of screening, yielding 47 (3.3%) women with prevalent TB. Symptom screening among women without known TB had a sensitivity of 28% and specificity of 84%. The poor performance of symptom screening to identify women with TB suggests that other approaches may be needed for intensified case-finding to be effective for this population.

## Introduction

Symptom screening is the first step in the World Health Organization (WHO)-recommended tuberculosis (TB) intensified case-finding (ICF) algorithm for people living with HIV. Multiple studies have evaluated the sensitivity of symptoms for identifying TB among HIV-infected individuals. Nine of these studies provided patient-level data on 8,148 patients with 495 TB diagnoses for a meta-analysis that calculated a combined sensitivity of 79% (the sensitivity was 90.1% when limiting the analysis to participants already in clinical care) and specificity of 50% when using a four-part symptom screen of any of cough, fever, night sweats, or weight loss [1]. Thus symptom screening is useful for identifying individuals with heightened likelihood of having TB to focus laboratory testing. It is also useful for identifying individuals with low likelihood of having TB to allow for accelerated initiation of antiretroviral therapy (ART) and TB preventive therapy [2], [3].

Symptom screening is also encouraged by the WHO for HIV-infected women receiving care during prenatal clinic visits. Diagnosing and treating pregnant women for TB is important because TB is a leading cause of maternal mortality in high HIV prevalence settings and maternal TB has negative effects on the fetus and infant including premature birth, low birth weight, and congenital or neonatal TB infection or disease [4]–[10]. However, data are limited on the optimal approach to ICF in this population, including the utility of symptom screening. Despite limited data on symptom screening when used for HIV-infected pregnant women, the WHO guidelines recommend the four-part symptom screen for this population [3]. Subsequent to the publication of the WHO guidelines, a study of pregnant women from India suggested low sensitivity of the four-part symptom screen when used alone [11]. However, lack of a gold-standard was a limitation of that study [12]. We sought to assess the prevalence of pulmonary TB and evaluate the validity of the WHO four-part symptom screen among HIV-infected pregnant women in the Matlosana sub-District in South Africa.

## Methods

This research was conducted according to the principles expressed in the Declaration of Helsinki; written informed consent was obtained from all participants prior to study procedures. Approvals of the study and consent process were received from the Johns Hopkins University and the University of the Witwatersrand. We prospectively recruited consecutive, HIV-infected pregnant women who had tested HIV seropositive at a prior prenatal clinic visit, were at least 18 years of age, and had been aware of their HIV status for at least 7 days. We included the latter criterion to mitigate negative responses to questions regarding TB symptoms from women who had just learned their HIV test results [13]. In this sub-district, symptom screening was not systematically used for pregnant women; when symptoms were investigated, sputum smear was the only test used. Sputa were not sent for culture prior to this implementation research project. Recruitment occurred on rotating clinic days at prenatal clinics at 16 primary healthcare centers and the regional hospital located in a peri-urban region of South Africa. This region has approximately 480,000 inhabitants residing in a town, five residential townships, and on large farms. Women, who consented, first provided expectorated sputa which were rated by a study nurse as ‘good sputum’ or ‘saliva.’ Following obtaining sputum, we conducted a brief interview that included questions related to demographics, pregnancy, current and prior TB treatment, TB exposure (father of the fetus currently or someone at home in the past five years), and TB symptoms. TB symptom questions included the presence of current cough, sputum production, fever, weight loss, and night sweats, as well the duration of each symptom.

Sputum specimens were subjected to both fluorescence microscopy using auramine-rhodamine staining and mycobacterial culture using the BACTEC MGIT 960 System (Becton Dickenson, United States). Those that were positive for *Mycobacteria* species had *M. tuberculosis* confirmed from the culture isolate with the Hain GenoType MTBDRplus system or the Hain GenoType Mycobacterium CM PCR (Hain Lifescience GmbH, Germany).

We defined *previously undiagnosed prevalent TB* as sputum culture positive for TB and not currently on TB therapy. We defined *diagnosed prevalent TB* as being on TB therapy at the time of enrollment. Total and previously undiagnosed prevalent TB were reported with 95% confidence intervals using the exact method. Logistic regression was used to assess associations with all prevalent TB and participants' characteristics (excluding symptoms). Sensitivity and specificity and positive and negative predictive values of symptom combinations for previously undiagnosed prevalent TB were calculated with 95% confidence intervals using the efficient-score method corrected for continuity [14]. We assessed performance of individual symptoms and the four-symptom screen comparing any one of the four symptoms to none of the four symptoms.

## Results

During the period from May 2010 to December 2011, of 5,251 pregnant women attending 17 antenatal clinics, 1,515 (29%) were found to be HIV-infected, of whom 1,451 (96%) were enrolled. Symptom data were missing from one and sputum culture was contaminated or culture data were missing for 35; these women were excluded from further analyses.

The median age of the remaining 1,415 women was 27 years (interquartile range (IQR): 23, 32), their median gestational age was 24 weeks (IQR: 18, 28), and the most recent median CD4 count was 394 cells/ µL (IQR: 271, 533) with 180 (13%) having CD4 counts ≤200 cells/ µL and 556 (40%) having CD4 counts <350 cells/ µL (Table 1). Most women (74%) were attending their second or third antenatal clinic visit at the time of recruitment. Twelve women (0.8%) were already on TB treatment at enrollment and were excluded from the symptom sensitivity and specificity analyses. Among women *not* on TB treatment, cough was reported by 110 (8%) with 66 reporting that they were producing sputum (5%), fever was reported by 51 (4%), night sweats by 49 (3%), and weight loss by 103 (7%). The median weight gain from the prior prenatal care visit for women in the second and third trimesters was 0.36 kg/week (IQR: 0, 0.87). A measured weight loss from the prior prenatal visit of >1 kg was recorded for 120 (8%) women; 26 of whom also self-reported weight loss. Any one of the four TB symptoms was reported by 226 women (16%).

**Table 1:** Characteristics of participants.

Characteristic	All	Without TB*	With previously undiagnosed TB*
	N(%), median (IQR)	N(%), median (IQR)	N(%), median (IQR)
Population	1415	1368	35
Maternal age, median, years	27 (23, 32)	27 (23, 32)	29 (25, 33)
Gestational age, median, weeks	24 (18, 28)	24 (18, 28)	22 (18, 28)
Weight at screening, median, kg	68 (60, 80)	68 (60, 80)	68 (60, 81)
Measured weight change, median, kg/wk	0.36 (0, 0.87)	0.37 (0, 0.87)	0.095 (−0.22, 0.53)
Ever had TB before (yes)	111 (7.8)	109 (8.0)	1 (2.8)
TB treatment at screening (yes)	12 (0.85)	-	-
Anyone at home with TB in past 5 years (yes)	351 (25)	332 (24)	12 (34)
Child's father currently has TB (yes)	11 (0.78)	11 (1.0)	0 (0)
Mother smokes (yes)	40 (2.8)	38 (2.8)	2 (5.7)
Hemoglobin at screening, median, mg/dL	11.2 (10, 12)	11.3 (10, 12.5)	10.5 (10, 11.2)
CD4 count, most recent, median, cells/ µL	394 (271, 533)	397 (275, 536)	261 (143, 409)
CD4 count group, most recent, cells/ µL			
≤200	180 (13)	165 (12)	13 (37)
201–350	383 (27)	367 (27)	11 (31)
>350	825 (58)	809 (59)	11 (31)
Missing	27 (1.9)	27 (2.0)	0 (0)
Antiretroviral management, at screening			
AZT pMTCT	856 (60)	832 (61)	20 (57)
cART	2 (0.14)	2 (0.1)	0 (0)
No ART agent	557 (39)	534 (39)	15 (43)
Symptoms			
Cough	115 (8.1)	102 (7.4)	8 (23)
Fever	52 (3.7)	50 (3.6)	1 (2.8)
Weight loss	107 (7.6)	100 (7.3)	3 (8.6)
Night sweats	51 (3.6)	45 (3.3)	4 (11)

pMTCT: prevention of mother-to-child transmission; AZT: zidovudine; cART: combination antiretroviral therapy; ART: antiretroviral therapy

* excluding diagnosed prevalent TB

Sputum samples from the 1415 women were rated as ‘good sputum’ from 228 (16%) and as ‘saliva’ from 1187 (84%). Of all these samples, 3 were smear positive and 72 were culture positive. Of the culture positives, 39 were speciated as *Mycobacterium tuberculosis*, 9 as *Mycobacterium avium* complex, and 24 as other *Mycobacterium* species. Two women had isoniazid mono-resistance and two had multidrug resistant TB. Of those with *M. tuberculosis* on culture, 4 were already on TB treatment. Thus 35 had previously undiagnosed prevalent TB disease (2.5%; 95% confidence interval [CI]: 1.7, 3.4). Adding the 12 women already on TB treatment provides a point prevalence for TB disease of 47/1415 (3.3%, 95% CI: 2.4, 4.4). None of the women were receiving isoniazid preventive therapy at the time of screening.

One participant with a positive sputum smear also had sputum that was culture positive for TB, the other two with positive sputum smears were culture negative and were not included as TB cases. The recorded quality of the sputum (good sputum versus saliva) was not associated with the prevalence of TB positivity (Fisher's exact p = 0.5) nor was it associated with the presence of symptoms (Fisher's exact p = 0.2).

In logistic regression only two (non-symptom) participant characteristics were associated with prevalent TB: lower CD4 count and self-reported exposure to a TB case (either at home in the past five years and/or the father of the fetus with current prevalent TB). In univariable analysis, compared to a CD4 count >350 cells/ µL the odds ratio for prevalent TB with CD4 count ≤200 cells/ µL was 4.6 (95% CI: 2.2, 9.5) and for 201–350 was 2.2 (95% CI: 1.1, 4.4). The odds ratio for prevalent TB when there was a reported exposure to a TB case in the past five years was 2.1 (95% CI: 1.2, 3.8). The odds ratios did not differ in multivariable analysis with CD4 count and household TB contact. All other recorded individual characteristics including age, hemoglobin, prior TB, and maternal smoking were not associated with prevalent TB (p all >0.1).

The majority of women with previously undiagnosed prevalent TB were asymptomatic. For those with symptoms, the medium reported duration of cough was 10 days (IQR: 4, 28); fever, 21 days (IQR: 7, 28); weight loss, 28 days (IQR: 14, 56); and night sweats, 21 days (IQR: 7, 28). The sensitivity of any one of the four symptoms, versus no symptoms, for TB disease was 28% (95% CI: 15, 46), specificity was 84% (95% CI: 82, 86), the positive predictive value was 4.4% (95% CI: 2.2, 8.2), and the negative predictive value was 98% (95% CI: 97, 98). Cough, irrespective of duration, was the most sensitive of the symptoms with a sensitivity of 23% (95% CI: 11, 40) (Table 2). The sensitivity of the four-part symptom screen was not affected by CD4 count (Table 2), by ART agent receipt, or TB exposure status (data not shown).

**Table 2:** Characteristics of symptom screening for TB among HIV-infected pregnant women.

Symptom	Total symptomatic	Total asymptomatic	Specificity	Sensitivity	Negative predictive value	Positive predictive value
	(TB/not TB)	(TB/not TB)	% (95% CI)	% (95% CI)	% (95% CI)	% (95% CI)
Cough	8/102	27/1266	92 (91, 94)	23 (11, 40)	98 (97, 98)	7.3 (3.4, 14)
Fever	1/50	34/1318	96 (95, 97)	2.8 (1.5, 17)	97 (96, 98)	1.9 (0.10, 12)
Weight loss	3/100	32/1268	93 (91, 94)	8.6 (2.2, 24)	98 (96, 98)	2.9 (0.76, 8.9)
Measured weight loss >1 kg	4/116	31/1252	92 (90, 93)	11 (3.7, 28)	98 (96, 98)	3.3 (1.1, 8.8)
Night Sweats	4/45	31/1323	97 (96, 98)	11 (3.7, 28)	98 (97, 98)	8.2 (2.6, 20)
CFSW*	10/216	25/1152	84 (82, 86)	28 (15, 46)	98 (97, 98)	4.4 (2.2, 8.2)
CFSW* CD4<350	7/104	17/421	80 (76, 83)	29 (13, 51)	96 (94, 98)	6.3 (2.8, 13)
CFSW* CD4≥350	3/106	8/710	87 (84, 89)	27 (7.3, 61)	99 (98, 99)	2.8 (0.7, 8.4)

* any one of cough, fever, night sweats, or weight loss (WHO recommended four-part symptom screen)

## Discussion

We found a high prevalence of TB (3.3%) among HIV-infected pregnant women in South Africa. We also identified a major limitation of symptom-based ICF among HIV-infected pregnant women: the majority of women with culture positive TB (73%) did not report any of the symptom components of the WHO four-part symptom screen - cough, fever, night sweats, or weight loss. Our TB prevalence is consistent with the combined HIV and TB epidemic in the region, although it is almost three-fold higher than the overall prevalence for South Africa of 1.3% [15]. Our TB prevalence estimate is also higher than reported from two studies of pregnant women from Soweto, South Africa in which TB disease was identified among 0.68–2% of HIV-infected pregnant women [13], [16]. However, sputum culture was only obtained from symptomatic women (23% and 32% of the participants from the respective studies) possibly explaining lower TB prevalence.

A possible explanation of the low sensitivity of symptom screening is physiological changes associated with pregnancy that may mask symptoms of TB, especially weight loss. However, symptom screening has also performed poorly in other settings, not involving pregnant women, including an ART treatment cohort in South Africa (sensitivity of 23% [17]), a Zambian HIV-infected prison population (sensitivity of 54% [18]), mine workers in South Africa (sensitivity of 31% [19]), and a community survey in South Africa (sensitivity of 33%, [20]) Other studies, including several of the studies in the WHO meta-analysis have reported much higher sensitivity [21]–[26]. It is unclear why there is such heterogeneity in sensitivity of the four-part symptom screen.

The poor performance of symptom screening is of major concern as asymptomatic, culture positive adults can transmit TB. In addition, asymptomatic, or sub-clinical, TB is well described to progress to symptomatic disease [27], [28] and timely diagnosis may be particularly important to prevent morbidity and mortality in pregnant women and their babies. We also observed poor performance of sputum fluorescence microscopy to identify TB disease: only 1 of 35 TB cases was positive by smear microscopy. Poor performance of smear microscopy has also been reported from other ICF programs [29], [30].

We noted a remarkably high proportion of women with self-reported TB contacts, either in their household or TB in the fetus's father; 25% of the women reported having a contact with TB in the past five years. We believe that this is consistent with the TB epidemic in South Africa, especially in poor peri-urban communities. With a median household size of approximately 5 people, over a time frame of 5 years, approximately 1% of household members would need to be diagnosed with TB each year to reach 25%. This is consistent with the annual TB incidence for South Africa of 993 per 100,000 population [15] and our own data describing rates of TB in households in the same area [29].

Our study has several strengths including using a population-based sample recruited during routine prenatal care and obtaining sputum from all participants. Limitations include potential incomplete symptom reporting by participants. However, we believe that performing the screening a week after HIV diagnosis and using trained study nurses may have improved symptom reporting to a level better than seen in routine clinical care. Another limitation is lack of data on the clinical progression of women with positive TB cultures as all the women in our study with a positive culture were referred for TB treatment. Finally, although liquid sputum culture is considered the ‘gold standard’ for TB diagnosis, cross-contamination could have occurred leading false-positive TB results. We believe that this is unlikely to have markedly influenced our results because (1) within our study, positive sputum cultures were distributed over time and did not cluster and (2) we used an accredited TB laboratory that uses quality control that includes monitoring for potential clusters that could suggest cross-contamination.

These results raise the question of whether the WHO-recommended approach of symptom screening is appropriate for pregnant women. Failure to diagnose TB has clear consequences for disease progression as well as potential consequences should ART be initiated. “Unmasking TB” can occur when symptoms develop shortly after ART initiation and present with severe illness which may be especially detrimental to a pregnant women or to her developing fetus [31]. The role of newer diagnostic technologies has not been evaluated among pregnant women but the Cepheid GeneXpert MTb/Rif may have a role [32]. The urine lipoarabinomannan enzyme-linked assay is unlikely to add sensitivity in a population whose median CD4 count is at least 100 cells/ µl higher than the HIV-infected adults in whom the lipoarabinomannan enzyme-linked assay has been found to be most useful (<200 cells/ µL) [33], [34]. Whether or not newer diagnostic technologies are found to be useful, we believe the four-part symptom screening is not an effective way to identify TB for TB treatment or to rule-out TB for ART or IPT initiation. Closer monitoring of pregnant women starting ART and further training of care providers in diagnosing and managing unmasking TB may be reasonable until improved screening techniques are available.

We conclude that, in this population of pregnant women, the WHO four-part symptom-screen was ineffective at identifying most cases of TB. Screening techniques with improved sensitivity or lower cost diagnostic tests that can be widely applied are urgently needed to address TB amongst HIV-infected pregnant women in resource limited settings.
